# Supplementary material for: A Hidden Chemical Assembly Mechanism: Reconstruction‐by‐Reconstruction Cycle Growth in HKUST‐1 MOF Layer Synthesis
Source: Chemphyschem. 2025 Feb 25;26(9):e202400968. doi: 10.1002/cphc.202400968 (PMC12058237; doi:10.1002/cphc.202400968)
Supplement: Supplementary file 1 — Supporting Information [file CPHC-26-e202400968-s001.pdf]

ChemPhysChem

Supporting Information

A Hidden Chemical Assembly Mechanism: Reconstruction-by-Reconstruction Cycle Growth in HKUST-1 MOF Layer Synthesis

T. Koehler, J. Schmeink, M. Schleberger, and F. Marlow*

Supplementary Information to:

A Hidden Chemical Assembly Mechanism: Reconstruction-by-Reconstruction Cycle Growth in HKUST-1 MOF Layer Synthesis

T. Koehler[a], J. Schmeink[b], M. Schleberger[b,c], F. Marlow*[a,c]

[a] Max-Planck-Institut für Kohlenforschung, Kaiser-Wilhelm-Platz 1, 45470 Mülheim an der Ruhr, Germany
[b] Fakultät für Physik, Universität Duisburg-Essen, Lotharstrasse 1, 47057 Duisburg, Germany
[c] Center for Nanointegration Duisburg-Essen (CENIDE), Carl-Benz-Str. 199, 47057 Duisburg, Germany

*Corresponding author (marlow@mpi-muelheim.mpg.de)

Content

1) The HKUST-1 lattice, layer structure, and surface steps 1
2) Literature on thickness increase per cycle and synthesis times 3
3) Atomic force microscopy 4
4) HPLC of the purging solution 7
5) Calculation of the effect of cross-contamination 9
6) Estimation of scattering effects 10
7) Reproducibility of the samples 11
8) Picture of the experimental setup 12
9) References 13

1) The HKUST-1 lattice, layer structure, and surface steps

The process of SURMOF synthesis is frequently designated as "layer-by-layer" synthesis. Nevertheless, the precise meaning of the term “layer” in the context of SURMOFs is seldom explicitly defined, contributing to some degree of confusion in the existing literature.

In this context, we utilize the term “layer” to describe the material added during one deposition cycle with both reactants in an idealized process of building-up the lattice on pre-defined positions. This leads to subsequent sub-layers enriched with one of the reactants. Based on that, the term layer thickness is defined as the distance between adjacent sub-layers of the same type, which are clearly separated by the sub-layer of the second type in the direction of the building-up. [1]

The layer thickness of HKUST-1 is illustrated in Fig. S1 for growth in the two most common cystallographic directions: 100 and 111. It is evident that the copper SBUs of the A_1 and A_2 layers cannot be deposited in a single step in the absence of BTC and therefore must belong to separate layers. In the case of the 100

direction, one unit cell contains four layers, with the layer thickness thus being $a/4$ = 0.65 nm. In the case of growth in the 111 direction, the layers are again clearly separated. The distance between adjacent layers is $(a\sqrt{3})/6$ = 0.76 nm because in this direction the unit cell contains 6 layers.

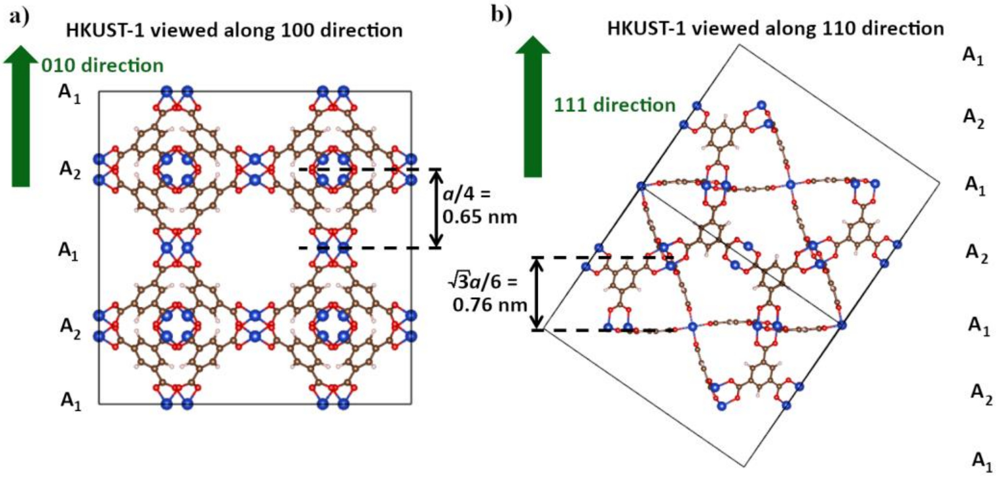

Fig. S1. The structure of HKUST-1 viewed a) along the 100 direction and b) along the 110 direction. In both cases, alternating A_1 and A_2 copper layers are observed, clearly separated by trimesic acid linkers. The distance between neighboring layers has been marked for both crystal directions. Atom colors: copper - blue, oxygen - red and carbon – brown, hydrogen - light grey. Atom positions from Ref.[2] illustrated via VESTA.

Larger values of possible layer thickness (1.1 – 1.5 nm) are sometimes reported in the literature (e.g. Ref. [3]). They are assigned to the dimensions of single secondary building units (SBU), preformed in solution. However, this assignment neither has an experimental basis nor can be found in the quoted literature (e.g. Ref. [4]). These values may cause confusion.

It is also interesting to compare measured surface steps[4] with the layer thickness since the layer structure of the lattice should be reflected here as well. The surface steps occur preferentially during one-pot syntheses, and can be avoided in stepwise procedures.

In Ref. [4], surface step heights of 1.5 nm were predominantly observed (after growth in 111 direction).[1, 4, 5] However, steps of 0.8 nm, 2.2 nm and 3.0 nm were also found.[1] These values are all multiples of the smallest possible atomic layer distance ($d_{111}/2$) containing complete SBUs, as seen in Figure 3b of Ref. [4]. The different Cu layers are not identical, but have differently oriented SBUs and different densities of SBUs. The reason that many step heights of twice the minimal amount were observed, is that surface termination of one layer is energetically favoured over the other, as discussed by Ref.[1]. Possibly, the double-layer height of one surface step has resulted in a misassignment of d_{111} as the smallest possible HKUST-1 layer thickness in parts of the HKUST-1 literature as opposed to the smaller value of $d_{111}/2$. Values in the range of 1.1 nm are probably due to a similar understanding of layer distances for HKUST-1 synthesized along the 100 direction.

2) Literature on thickness increase per cycle and synthesis times

An overview of experimentally observed thickness increases of HKUST-1 thin films per cycle d/n in the literature is provided in Table S1. Only those works were included that have proven a linear growth regime, which requires the measurement of multiple samples synthesized under the same conditions, but different cycle numbers, or the measurement of the thickness during the synthesis.

Table S1. Experimentally observed d/n values reported in the literature, as well as the deposition and thickness determination method.

Source	Synthesis method	*d*-determination	d/n [nm]
Munuera et al.[6]	Dip-coating	AFM	1.3
Arslan et al.[7]	Spray-coating	XRD peak width	10
Liu et al.[8]	Spray-coating	AFM	~7
Zhao et al.[9]	Dip-coating	TEM	3
Han et al.[10]	Vacuum growth	AFM	20
Ohara et al.[11]	Dip-coating	AFM	2-2.5
Chen et al.[12]	Spray-coating	SEM	2-2.5
Li et al.[13]	Spray-coating	AFM	~8
Stavila et al.[3]	LBL flow cell	QCM	2.8
Dhanapala et al.[14]	Dip-coating	Ellipsometry	0.7-1.4
	Spray-coating		2.1

An overview over experimentally observed Root mean squared (RMS) roughness values of HKUST-1 thin film surfaces and the immersion times of the stepwise synthesis procedure of a selection of literature works.

Table S2. Typical film properties and immersion times for thick LbL HKUST-1 films, synthesized via dip-coating.

Ref.	RMS roughness / nm	Immersion time in Cu / in BTC in min
Munuera et al. [6]	~ 5	30 min / 60 min
Liu et al. [7]	-	15 min / 30 min
Zhao et al. [8]	-	5 min / 5 min
Ohara et al. [9]	10 - 16	5 min / 5 min
Dhanapala et al. [10]	13 - 57	30 min / 60 min
Müller et al. [11]	-	10 min / 15 min
Thürmer et al. [12]	-	7.5 / 15 min
Gu et al. [13]	6	10 min / 15 min
This work	6 - 8	0.5 min / 0.5 min

3) Atomic force microscopy

In addition to the material of the main work, here all 15 AFM images, height probability distributions for evaluation of the film thickness and a table with these determined thicknesses, as well as the ones determined from lines scans are provided. All images were processed with Gwyddion as detailed in the experimental part of the main work.

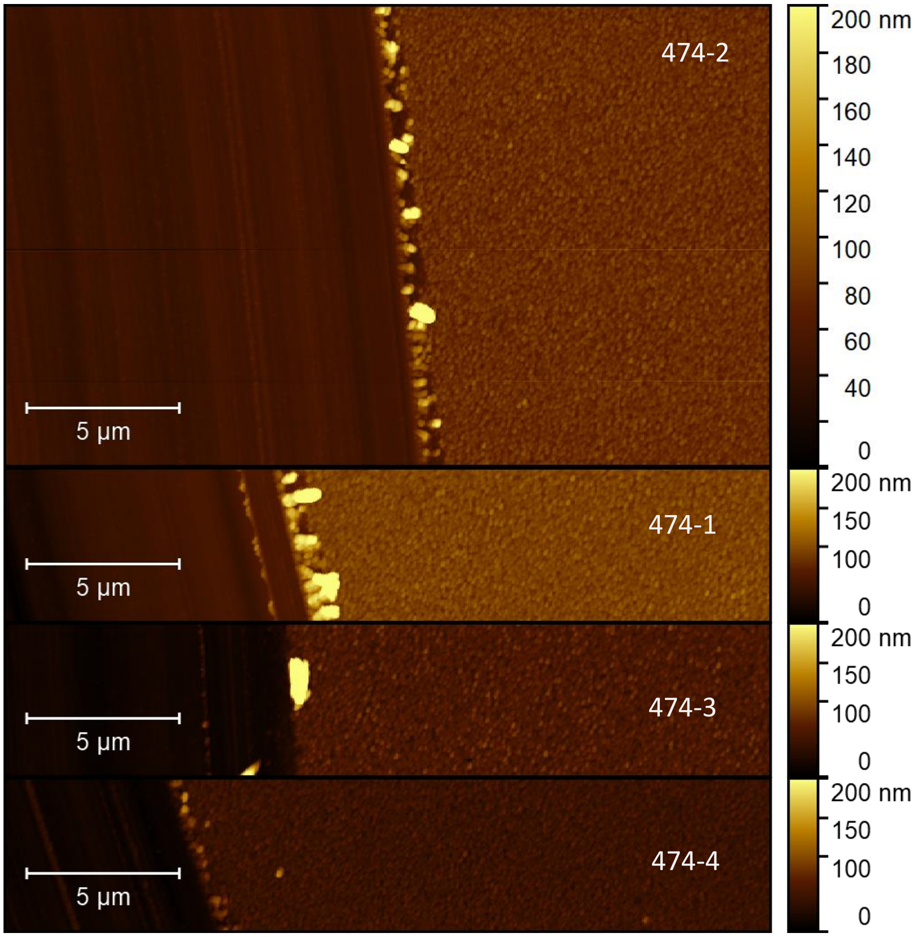

Fig. S2. AFM images of four HKUST-1 SURMOF samples after 10 synthesis cycles.

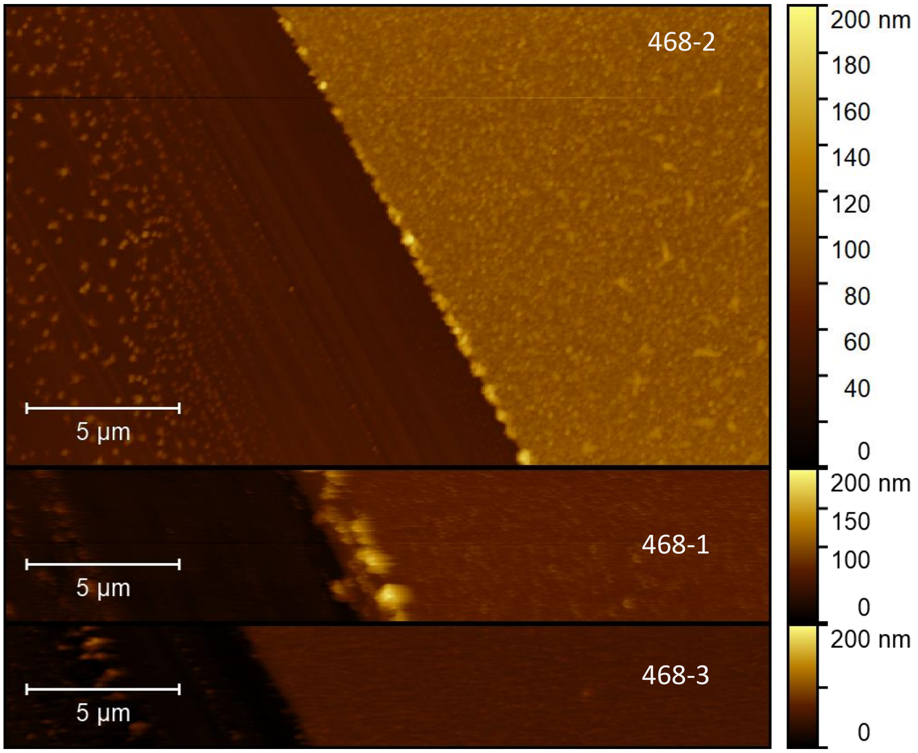

Fig. S3. AFM images of three HKUST-1 SURMOF samples after 20 synthesis cycles.

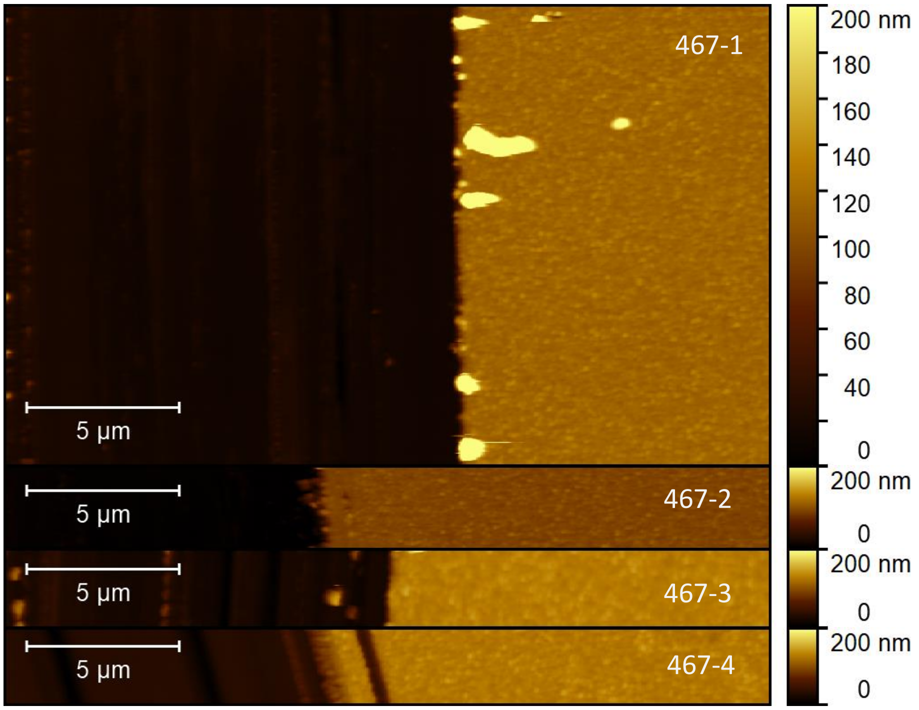

Fig. S4. AFM images of four HKUST-1 SURMOF samples after 40 synthesis cycles.

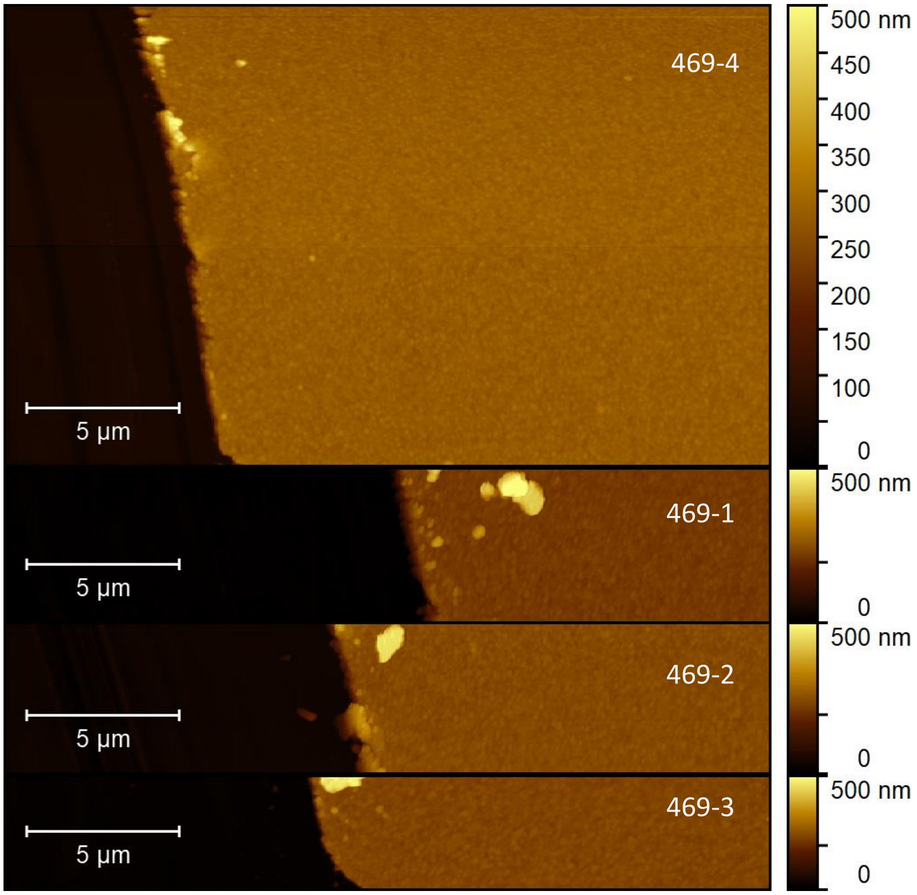

Fig. S5. AFM images of four HKUST-1 SURMOF samples after 80 synthesis cycles.

Table S3. The thickness *d*, determined from line scans, as well as from height distributions, RMS roughness for all samples, synthesized with different cycle numbers *n.*

n	sample	Linescan *d* [nm]	Height Distribution *d* [nm]	RMS roughness [nm]
10	474-1	23.7	26.0	7.7
10	474-2	20.7	20.8	7.6
10	474-3	36.1	31.1	9.8
10	474-4	22.2	23.0	6.9
20	468-1	57.2	61.2	5.8
20	468-2	49.5	46.9	6.6
20	468-3	45.1	44.9	7.0
20	468-4	-	-	-
40	467-1	102	102	5.2
40	467-2	91.7	101	5.2
40	467-3	109	119	6.9
40	467-4	97.5	110	4.9
80	469-1	213	222	7.4

80	469-2	216	218	7.2
80	469-3	218	210	8.7
80	469-4	219	219	8.6

4) HPLC of the purging solution

The purging solution 4 after 20 synthesis cycles was analyzed with High-Performance Liquid Chromatography (HPLC) in Fig. S6, as well as a reference sample of pure ethanol (Fig. S7) and a calibration measurement of BTC solution at a known concentration (Fig. S8). 2 µL of sample is dissolved in water with 0.1% trifluoroacetic acid and filtered through a separation column (YMC Triart C18, 50 mm, 1.9 µm, 0.5 mL/min, 30.8 MPa, 308 K). The change in UV transmission (G7117C 1260 Infinity II DAD HS, 220 nm) is measured at its bottom output.

The signal around 1.727 min can be clearly identified to belong to BTC. Based on the calibration measurement, the purging solution has a concentration of c = 1.337 µM.

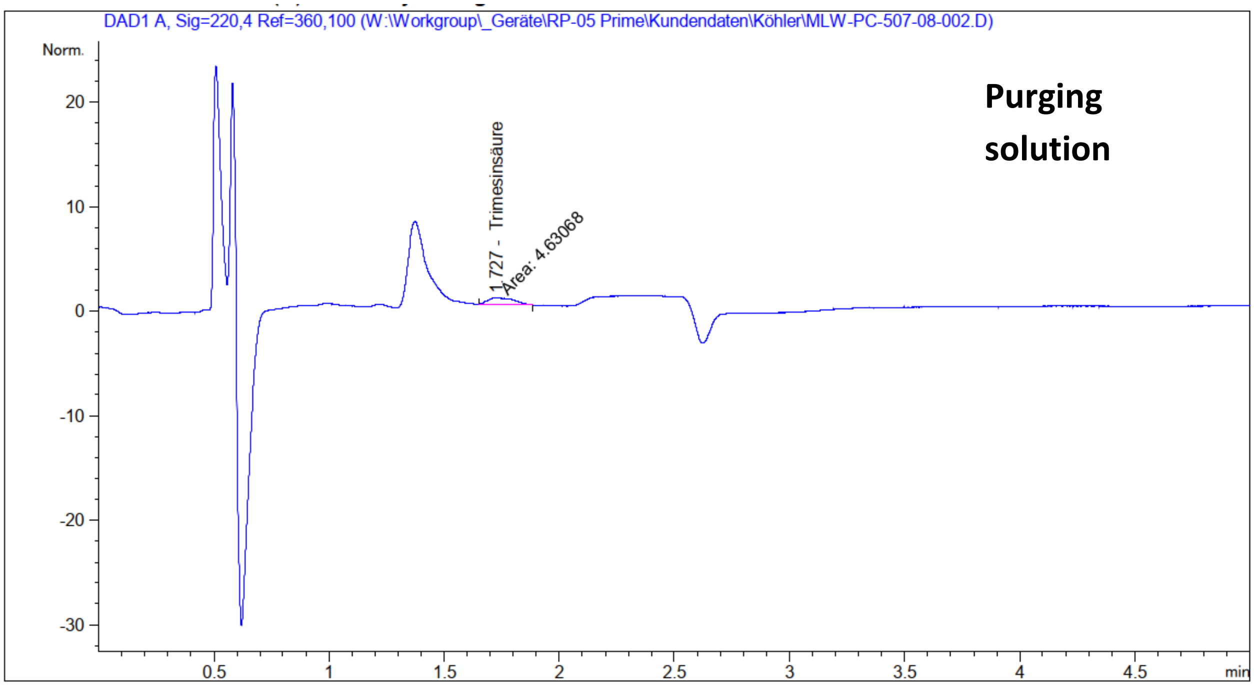

Fig. S6. UV/Vis signal at 220 nm as a function of time for solution 4 after 20 cycles of SURMOF synthesis.

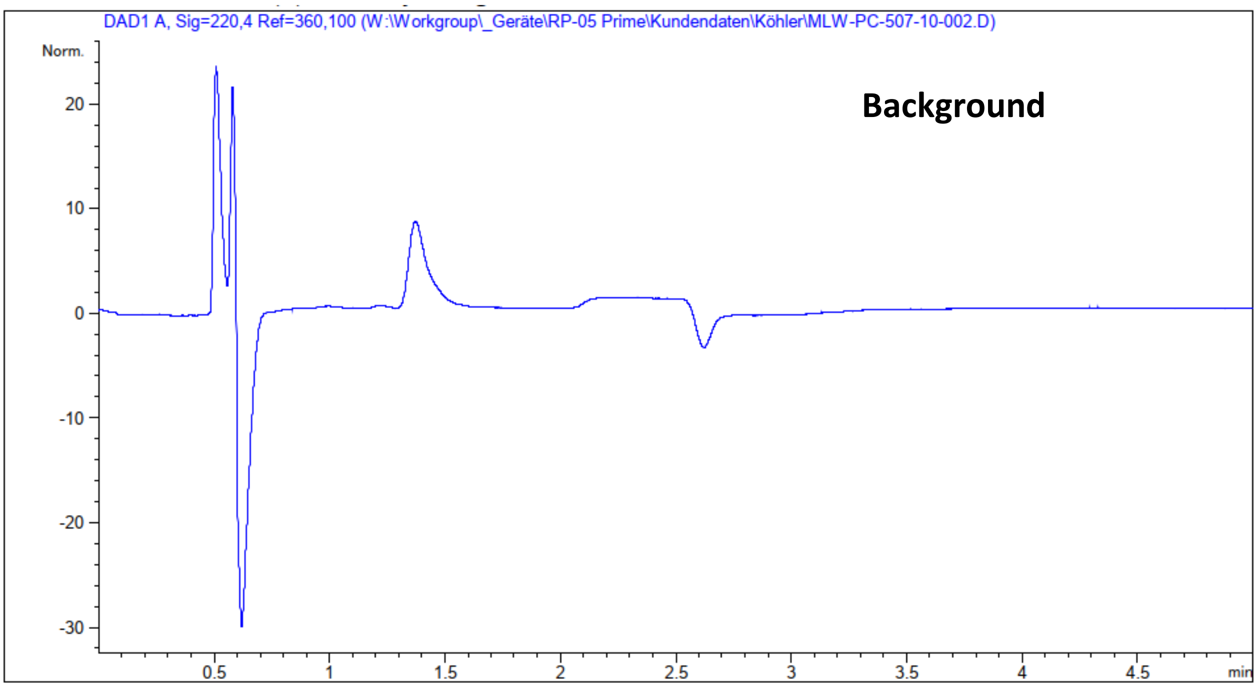

Fig. S7. Background measurement of pure ethanol.

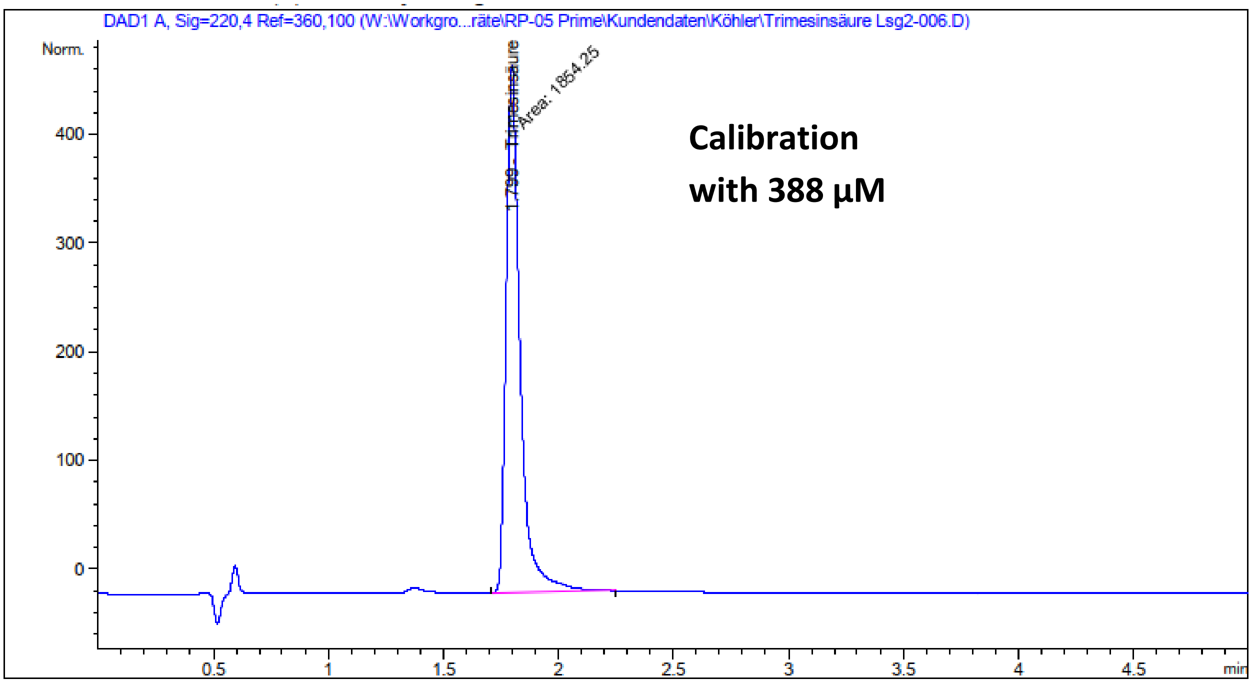

Fig. S8. Calibration measurement of BTC, dissolved at a concentration of 388 µM.

5) Calculation of the effect of cross-contamination

After 20 dip-coating cycles solution 4 ($V = 85$ mL) was contaminated with BTC at a concentration $c = 1.337$ µM, as determined from HPLC analysis. This corresponds to a total substance amount of

$$n_{BTC}(\text{sol. }4) = c_{BTC}(\text{sol. }4) \cdot V(\text{sol. }4) = 114\ \text{nmol.}$$

We assume that contamination increases linearly with each cycle. This will be the case, if:

1) The concentration of BTC in educt solution 3 ($c_{BTC} = 0.2$ mM) does not change significantly during the synthesis
2) The amount of substance transferred out of cleaning solution 4 into copper solution 1 is negligible compared to the one transferred into solution 4.

The total volume transferred from solution 3 to solution 4 during the whole 20 cycles is then

$$V(\text{sol. }3 \rightarrow \text{sol. }4) = \frac{n_{BTC}(\text{sol. }4)}{c_{BTC}(\text{sol. }3)} = \frac{c_{BTC}(\text{sol. }4) \cdot V(\text{sol. }4)}{c_{BTC}(\text{sol. }3)} = 0.568\ \text{mL}$$

or about 14.2 µL per sample transfer (V/40 due to 20 cycles and 2 sample holders). We believe 14.2 µL, or about one droplet, to be a very reasonable value for the utilized transfer procedure. Due to the same solvent and transfer procedure for each step, one can assume the transferred volume is similar between each of the solutions and therefore

$$V(\text{sol. }3 \rightarrow \text{sol. }4) = V(\text{sol. }4 \rightarrow \text{sol. }1).$$

We calculate the maximum amount of substance transferred from cleaning solution 4 into copper solution 1 per cycle and sample to be

$$n_{BTC}(\text{sol. }4 \rightarrow \text{sol. }1) = c_{BTC}(\text{sol. }4) \cdot \frac{V(\text{sol. }4 \rightarrow \text{sol. }1)}{40} = 19.0\ \text{pmol/transfer.}$$

Since this calculation is based on a BTC-concentration at the end of 20 cycles, this value should be understood more as an upper end. The BTC transferred during early cycles is even smaller than that.

This value also confirms the validity of our previous assumption 2 since

$$n_{BTC}(\text{sol. }4 \rightarrow \text{sol. }1) \ll n_{BTC}(\text{sol. }4).$$

$n_{BTC}(\text{sol. }4 \rightarrow \text{sol. }1)$ can now be compared to the substance amount needed for the growth of one monolayer. Looking at the crystal structure, we can estimate a linker concentration of 16 BTC per unit cell with a side view area in the HKUST-1 100-direction of $(2.6\ \text{nm})^2$. Sample dimensions are $A = 2$ cm x 2.5 cm (due to partial immersion in the solutions) with 2 samples back to back to each other, thereby requiring

$$N_{BTC}(\text{monolayer}) = 2 \cdot A \cdot \frac{16}{(2.6\ \text{nm})^2} = 2.37 \cdot 10^{15}\ \text{molecules}$$

or

$$n_{BTC}(\text{monolayer}) = \frac{N_{BTC}}{N_A} = 3.93\text{ nmol}$$

for growth of one monolayer. Since the transferred substance amount of BTC $n_{BTC}(\text{sol. }4 \rightarrow \text{sol. }1)$ is about 2 orders of magnitude smaller than the amount required per monolayer, we follow that the increase in growth rate (discussed in the main work) cannot be explained from cross-contamination of the solutions.

$$n_{BTC}(\text{sol. }4 \rightarrow \text{sol. }1) = 19.0\text{ pmol} \ll n_{BTC}(\text{monolayer}) = 3.93\text{ nmol}$$

6) Estimation of scattering effects

The synthesized MOF-films do not show any scattering effects in their visual appearance. In the transmission spectra, some scattering effects may be present, however, all the main features of the spectra could also be assigned to absorption and reflection effects. In this section, we estimate the magnitude of the scattering effects by an upper limit. This value may be useful to estimate the utility of the films in applications and to underline the exceptional optical quality of the films.

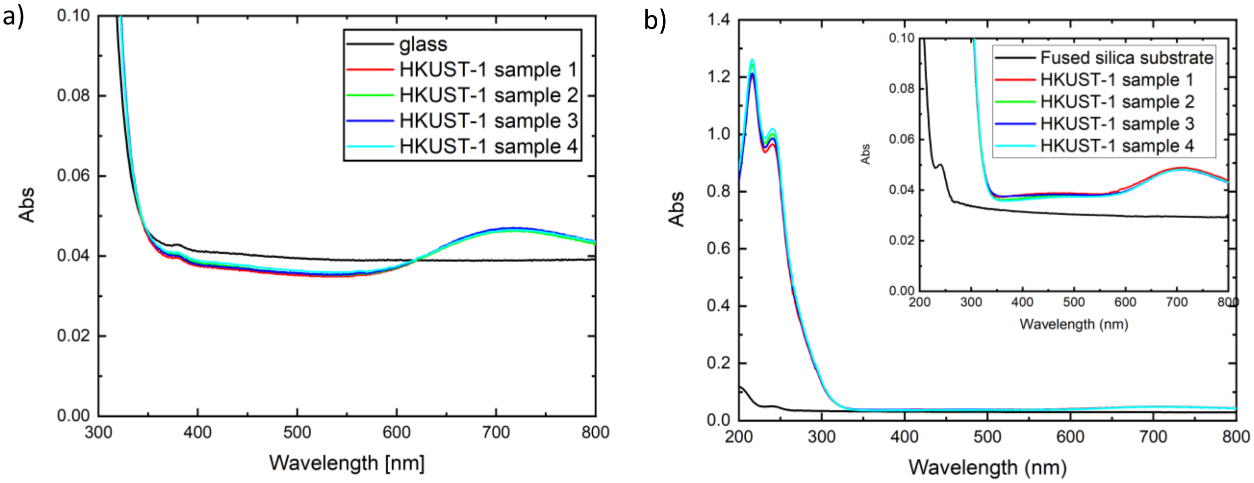

Fig. S9. UV/Vis measurements at 40-cycle samples on a) glass and b) on quartz glass. Four different samples are shown in each graph. Inset: magnification of the nearly transparent region.

As illustrated in Fig. S9, coating the substrates changes the transmission in the sub-% range in the visible part of the spectrum and to a greater extent in the UV. These changes are the absorption effects of the MOF lattice, the dd-transition of the Cu dimer, the modified reflection across the spectrum, and possibly the scattering. These effects, if present, should be most evident in the blue transparency window between 400 and 550 nm (blueTW).

Two observations allow for the estimation of scattering effects, (i) the wavelength dependent effects between 400 and 550 nm are small and (ii) only a part of the small effect on quartz glass can be attributed to potential scattering. Therefore, we estimate the scattering effects to be less than one half of the total effects in the blueTW (total effect = 0.003), leading to $S_{ca} \leq ½\ 0.003 /2.3 = 0.0006 = 0.06\ \%$. By chance, upper-bound estimates give the same value. This value is below the detection limit of many measurement

devices and below the observation threshold under normal observation conditions. It can be used to estimate the possible application possibilities of the films.

7) Reproducibility of the samples

The reproducibility of the samples is visible in UV/vis an FTIR investigations. Fig. S10 shows groups of spectra arising from four independent samples. They are nearly identical.

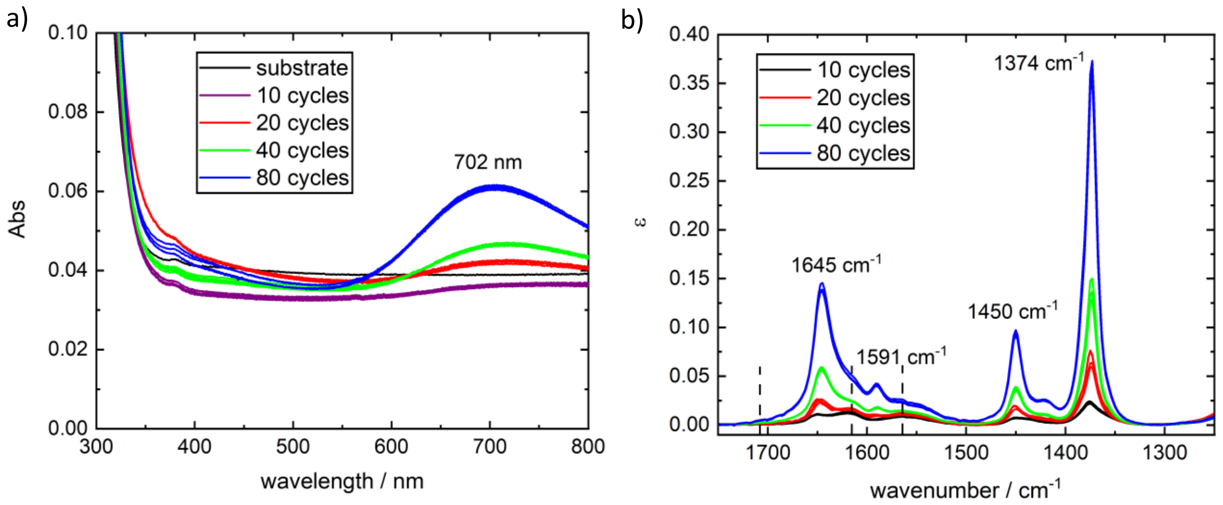

Fig. S10. a) UV/Vis and b) FTIR spectra of four HKUST-1 SURMOF samples synthesized each with 10, 20, 40 and 80 cycles, respectively. The dashed lines in b indicate defect peak positions.

8) Picture of the experimental setup

An image of the dip-coating holder is shown in Figure S11. It enables quick solution exchange and a safe handling of the samples especially during the tilting process described in the main text.

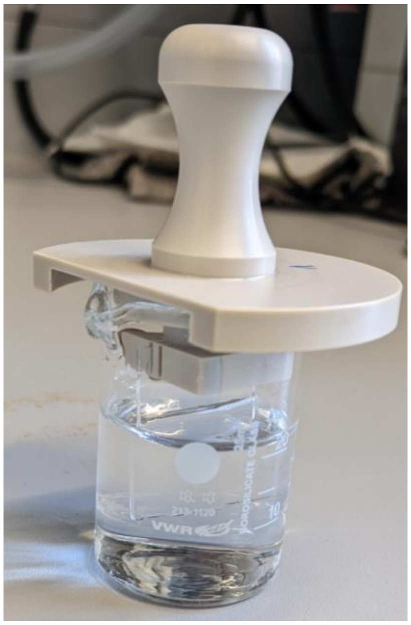

Figure S11. Glass substrates hanging from the samples holder, which is placed on top of a 20 mL beaker filled with one of the reactant solutions.

9) References

[1] M. Shöâeè, M. W. Anderson, M. P. Attfield, *Angew Chem Int Ed Engl* **2008**, *47*, 8525-8528.

[2] A. A. Yakovenko, J. H. Reibenspies, N. Bhuvanesh, H.-C. Zhou, *J. Appl. Crystallogr.* **2013**, *46*, 346-353.

[3] V. Stavila, J. Volponi, A. M. Katzenmeyer, M. C. Dixon, M. D. Allendorf, *Chem. Sci.* **2012**, *3*, 1531-1540.

[4] M. Shöâeè, J. R. Agger, M. W. Anderson, M. P. Attfield, *CrystEngComm* **2008**, *10*, 646-648.

[5] N. S. John, C. Scherb, M. Shoaee, M. W. Anderson, M. P. Attfield, T. Bein, *Chem. Commun.* **2009**, 6294-6296.

[6] C. Munuera, O. Shekhah, H. Wang, C. Wöll, C. Ocal, *Phys. Chem. Chem. Phys.* **2008**, *10*, 7257-7261.

[7] J. Liu, O. Shekhah, X. Stammer, H. K. Arslan, B. Liu, B. Schüpbach, A. Terfort, C. Wöll, *Materials* **2012**, *5*, 1581-1592.

[8] J. Zhao, B. Gong, W. T. Nunn, P. C. Lemaire, E. C. Stevens, F. I. Sidi, P. S. Williams, C. J. Oldham, H. J. Walls, S. D. Shepherd, M. A. Browe, G. W. Peterson, M. D. Losego, G. N. Parsons, *J. Mat. Chem. A* **2015**, *3*, 1458-1464.

[9] H. Ohara, S. Yamamoto, D. Kuzuhara, T. Koganezawa, H. Oikawa, M. Mitsuishi, *ACS Appl. Mater. Interfaces* **2020**, *12*, 50784-50792.

[10] B. D. Dhanapala, D. L. Maglich, M. E. Anderson, *Langmuir* **2023**, *39*, 12196-12205.

[11] K. Müller, K. Fink, L. Schottner, M. Koenig, L. Heinke, C. Wöll, *ACS Appl. Mater. Interfaces* **2017**, *9*, 37463-37467.

[12] K. Thurmer, C. Schneider, V. Stavila, R. W. Friddle, F. Leonard, R. A. Fischer, M. D. Allendorf, A. A. Talin, *ACS Appl. Mater. Interfaces* **2018**, *10*, 39400-39410.

[13] Z.-G. Gu, A. Pfriem, S. Hamsch, H. Breitwieser, J. Wohlgemuth, L. Heinke, H. Gliemann, C. Wöll, *Microporous Mesoporous Mater.* **2015**, *211*, 82-87.
